# Verifying the Geographical Origin and Authenticity of Greek Olive Oils by Means of Optical Spectroscopy and Multivariate Analysis

**DOI:** 10.3390/molecules25184180

**Published:** 2020-09-12

**Authors:** Renate Kontzedaki, Emmanouil Orfanakis, Georgia Sofra-Karanti, Katerina Stamataki, Aggelos Philippidis, Aikaterini Zoumi, Michalis Velegrakis

**Affiliations:** 1 Institute of Electronic Structure and Laser, Foundation for Research and Technology-Hellas (IESL-FORTH), 700 13 Heraklion, Crete, Greece; renate.kontz@windowslive.com (R.K.); manolis.orfanak@gmail.com (E.O.); gogosofra97@gmail.com (G.S.-K.); skaterin@iesl.forth.gr (K.S.); filagg@iesl.forth.gr (A.P.); azoumi@iesl.forth.gr (A.Z.); 2 Department of Chemistry, University of Crete, 700 13 Heraklion, Crete, Greece; 3 Department of Materials Science and Technology, University of Crete, 700 13 Heraklion, Crete, Greece

**Keywords:** olive oil, geographical origin, authenticity, visible spectroscopy, Raman spectroscopy, Partial Least Squares Discriminant Analysis, machine learning

## Abstract

Olive oil samples from three different Greek regions (Crete, Peloponnese and Lesvos) were examined by optical spectroscopy in a wide spectral region from ultraviolet to near infrared using absorption, fluorescence and Raman spectroscopies. With the aid of machine learning methods, such as multivariate partial least squares discriminant analysis, a clear classification of samples originating from the different Greek geographical regions was revealed. Moreover, samples produced in different subareas of Crete and Peloponnese were also well discriminated. Furthermore, mixtures of olive oils from different geographical origins were studied employing partial least squares as a tool to establish a model between the actual and predicted compositions of the mixtures. The results demonstrated that optical spectroscopy combined with multivariate statistical analysis can be used as an emerging innovative alternative to the classical analytical methods for the identification of the origin and authenticity of olive oils.

## 1. Introduction

Olive oil is a fruit juice produced in a completely natural way from olives (*Olea europaea*), without the use of chemical solvents or other additives. Its phenolic components have been shown to have anti-inflammatory and antioxidant properties [1]. The protective role of the phenolic components of olive oil is well known [2]. Thus, its consumption has been increasing worldwide. However, the increased demand of olive oil may lead to illegal activities, such as false declaration of geographical origin and counterfeiting authenticity.

In order to prevent such incidents, the European Union has enacted a series of regulations to attest olive oils [3]. The geographical indications of PDO (protected designation of origin), PGI (protected geographical indication) and TSG (traditional specialty guaranteed) are used for the commercial protection of a product originating from a specific region and following a traditional production process. PDO includes agricultural products that are produced in a specific geographical area. PGI includes products linked to a geographical area. More specifically, at least one of the production stages occurs in that area. TSG includes products that have been created using traditional materials or traditional production methods [4].

In previous years, many analytical techniques have been used to protect the authenticity of olive oils. These techniques include chromatography, nuclear magnetic resonance (NMR) spectroscopy and isotope ratio mass spectrometry [5]. NMR spectroscopy was used to investigate the authenticity of virgin olive oils from Italy and Greece [6]. Also, ^1^H high-field NMR was used to analyze olive oils collected in different Italian areas and to evaluate the potential of this technique for geographical characterization [7]. Moreover, authentic Italian olive oils were characterized by isotope ratios of C, H and O [8]. Additionally, measurements of δ^13^C and δ^18^O were performed on olive oil samples produced in Greece, Morocco, Spain, Italy, Tunisia and Turkey. The results showed a trend to classify samples according to geographical origin [9]. Chromatographic techniques, such as capillary electrochromatography, high performance liquid chromatography (HPLC) and gas chromatography-mass spectrometry (GC-MS), have been used to determine the geographical origin of olive oils. More specifically, Lopez-Feria et al. conducted a mass spectrometric study for the discrimination of extra virgin olive oils belonging to different PDOs [10]. In addition, olive oil samples from different European countries were successfully separated by proton transfer reaction mass spectrometry (PTR-MS) headspace analysis [11]. Recently, Mikrou et al. accomplished the varietal and geographical discrimination of Greek olive oils based on the chromatographic determination of fatty acids, squalene and tocopherols [12]. Even though analytical methods are quantitative and accurate, they are also sophisticated and time consuming.

Spectroscopic techniques have been used for the discrimination of olive oils based on their geographical origin. Visible Fourier transform infrared and near- and mid-infrared spectroscopies were used to classify olive oil samples based on their geographical origin. More specifically, Lin et al. reported the potential of visible and near-infrared spectroscopies for discriminating between extra virgin olive oils from Spain, Italy and Turkey [13]. Moreover, Downey et al. examined the ability of visible and near-infrared spectroscopies to discriminate extra virgin olive oils from the eastern Mediterranean [14]. In addition, Fourier transform infrared spectroscopy (FTIR) in combination with multivariate analysis can distinguish extra virgin olive oils from Greece, Italy, Portugal and Spain [15]. Gurdeniz et al. demonstrated the efficiency of mid-IR spectroscopy in the classification of Turkish olive oils according to their growing location [16].

The aim of this study was to analyze Greek olive oil samples using optical spectroscopic techniques, which are able to stand as an innovative alternative to conventional analytical techniques. Spectroscopic methods are of low cost, capable of providing information rapidly and need no special pretreatment of the samples.

## 2. Results and Discussion

**Figure 1:**
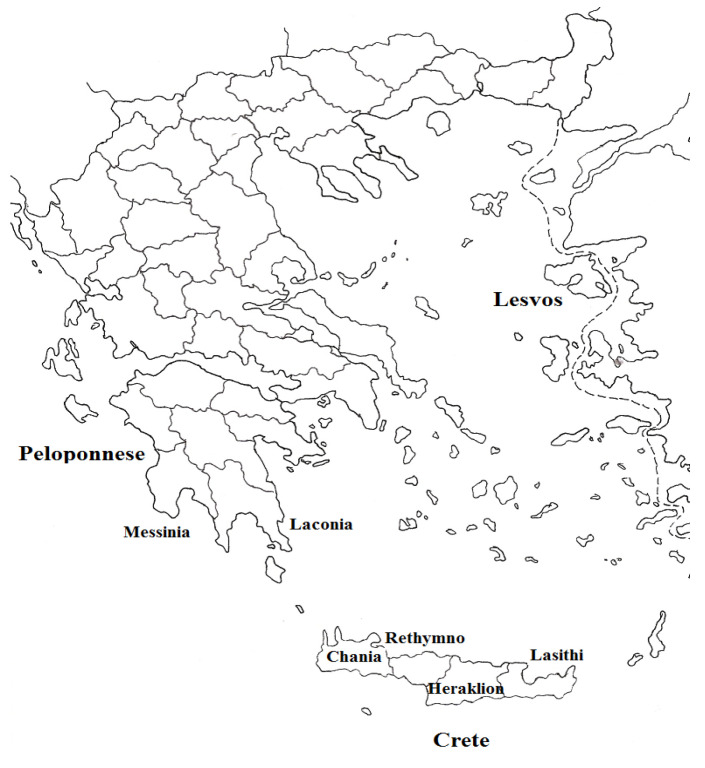
Map of Greece showing the geographical origin of the olive oil samples.

In this study, we investigated the classification of olive oil samples using optical spectra for various spectral regions from ultraviolet (UV) to near-Infrared (NIR), and also fluorescence and Raman spectroscopic data. Seventy-one samples of Greek olive oils were collected in the winter period of 2018–2019 from three regions of Greece, namely Crete, Peloponnese and Lesvos. These included 40 samples of Koroneiki variety produced in different subareas (prefectures) of Crete (16 from Heraklion, 7 from Lasithi, 8 from Rethymnon and 9 from Chania), 16 samples of Koroneiki and Kolovi varieties produced in different subareas (prefectures) of Peloponnese (12 from Messinia and 4 from Laconia) and 15 samples of Koroneiki, Kolovi and Adramitiani varieties from Lesvos. A map of the above-mentioned regions is shown in Figure 1.

Graphical results are presented only for the spectroscopic techniques that achieved optimal classification of the samples. Table 1 shows the percentage of successful classification of the olive oil samples by PLS-DA for all spectral regions under examination. The application of partial least squares discriminant analysis (PLS-DA) on the absorption (UV, visible and NIR), fluorescence and Raman spectra of the olive oils from different geographical origins demonstrated that visible and Raman spectroscopy are superior in comparison to other spectroscopic techniques for the purpose of this study.

**Figure 2:**
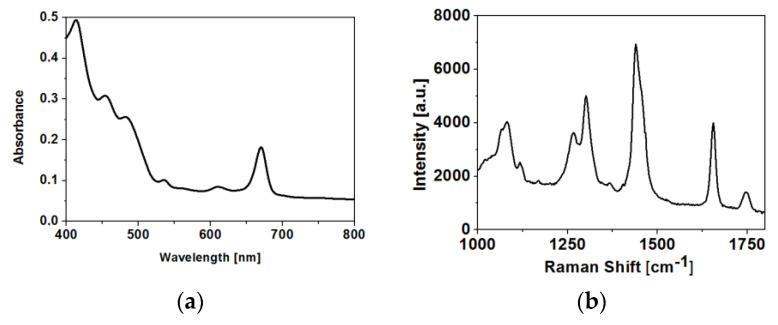
(**a**) Visible absorption spectrum and (**b**) Raman spectrum of olive oil.

A typical visible absorption spectrum of olive oil is shown in Figure 2a. Samples of olive oil present peaks from carotenoids at 454 and 480 nm, while peaks at 416 and 670 nm correspond to chlorophyll [17]. A typical Raman spectrum of olive oil from 1000 to 1800 cm^−1^ is shown in Figure 2b. The characteristic Raman bands correspond to the vibrations of fatty acids in olive oils. In particular, the band at 1082 cm^−1^ is due to C-C stretching vibrations, and the one at 1265 cm^−1^ is due to =C-H stretching vibrations. The bending vibrations of C-H is observed at 1300 and 1441 cm^−1^, while C=C stretching of unsaturated fatty acids is observed at 1657 cm^−1^. A weak band at 1747 cm^−1^ corresponds to C=O stretching of esters in olive oil [18,19,20,21].

**Figure 3:**
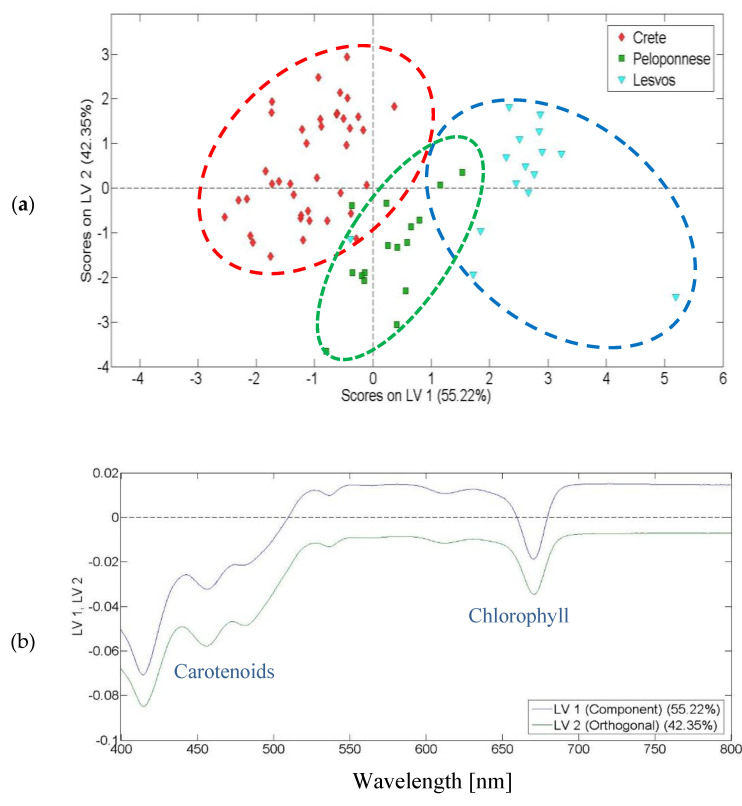
Classification of olive oils: (**a**) score plot for the discrimination of the olive oils from Crete, Peloponnese and Lesvos using visible absorption spectroscopy; (**b**) loading plot based on visible spectra.

The application of PLS-DA on the visible spectra brought on a clear separation of the olive oils produced in different geographical regions as shown in the score plot of Figure 3a. In total, 97.57% of data variance was explained by the first two latent variables (LV1 and LV2). The loading plot of Figure 3b depicts that both the first and the second latent variable played a notable role in the discrimination of the olive oils based on their geographical origin. Four main bands contributed to the discrimination, namely at 454 and 480 nm, that correspond to carotenoids, and at 416 and 670 nm, that correspond to chlorophyll. Therefore, chlorophyll and carotenoid contents are different in olive oils produced in different regions of Greece [22]. As mentioned by Mateos et al., differences in chlorophyll and carotenoid contents depend on the growing regions of the olive oils [23].

Table 2 shows the PLS-DA model accuracy based on visible spectra. The predicted values of the statistical model are satisfactory and only two samples were misclassified. A five latent variables model cross-validated with Venetian blinds (nine splits) was applied.

**Figure 4:**
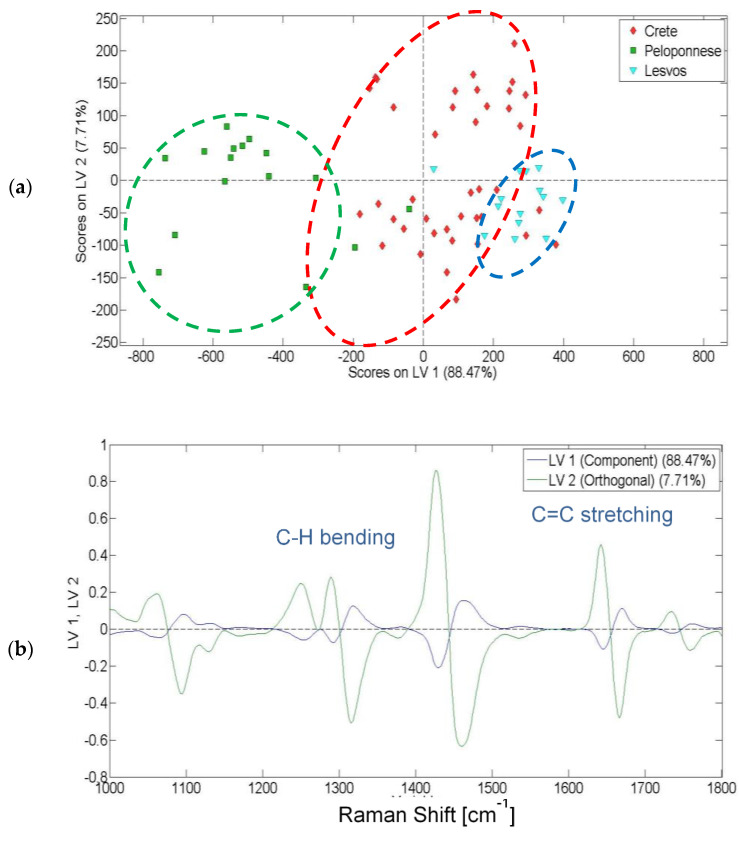
Classification of olive oils: (**a**) score plot for the discrimination of the olive oils from Crete, Peloponnese and Lesvos using Raman spectroscopy; (**b**) loading plot based on Raman spectra.

By using Raman spectroscopy, in a similar way to the visible absorption, the olive oil samples from Crete, Peloponnese and Lesvos were separated into three groups (Figure 4a). In total, 96.18% of data variance was explained by the first two latent variables (LV1 and LV2). It should be mentioned that the samples from Crete were further discriminated into two subgroups. Those subgroups correspond to the samples produced in western (negative values of y axis) and eastern Crete (positive values of y axis). The loading plot of Figure 4b depicts that the second latent variable played a notable role in the discrimination of the olive oils based on their geographical origins.

The four main bands that contributed to the discrimination, namely at 1082, 1300, 1441 and 1657 cm^−1^, corresponded to the vibrations of saturated and unsaturated fatty acids in olive oil. Consequently, Raman spectroscopy in combination with PLS-DA revealed a clear differentiation of fatty acid content with respect to geographical origin. Diraman et al. have used fatty acid composition of olive oils for the discrimination of geographical origin for various Turkish virgin olive oils [24]. Kosma et al. have also shown that variation in the fatty acid content of Greek olive oils can be attributed to different geographical origins [25]. In this study, the harvest time, crop altitude, irrigation scheme and fertilization of the samples were similar. Therefore, the differences in fatty acid content can be attributed to differences in the soil and environmental conditions of the regions (average temperature, rainfall and duration of sunshine). The average rainfall value from May to October 2018 was recorded at 52 mm for Chania and 1.67 mm for Lasithi. Similarly, the duration of sunshine was recorded at 327.67 h for Lesvos and 223 h for Lasithi [26]. Inglese et al. reported that environmental conditions affect both the fatty acid and the unsaponifiable fraction of the olive oils [27]. Fluctuations in the fatty acid content have been related to average temperature until harvest [28]. Additionally, Stefanoudaki et al. were able to classify olive oils according to their growing locations due to environmental factors such as rainfall [29].

Table 3 shows the PLS-DA model accuracy based on Raman spectra. A clear discrimination was accomplished. A six latent variables model cross-validated with Venetian blinds (nine splits) was applied.

**Figure 5:**
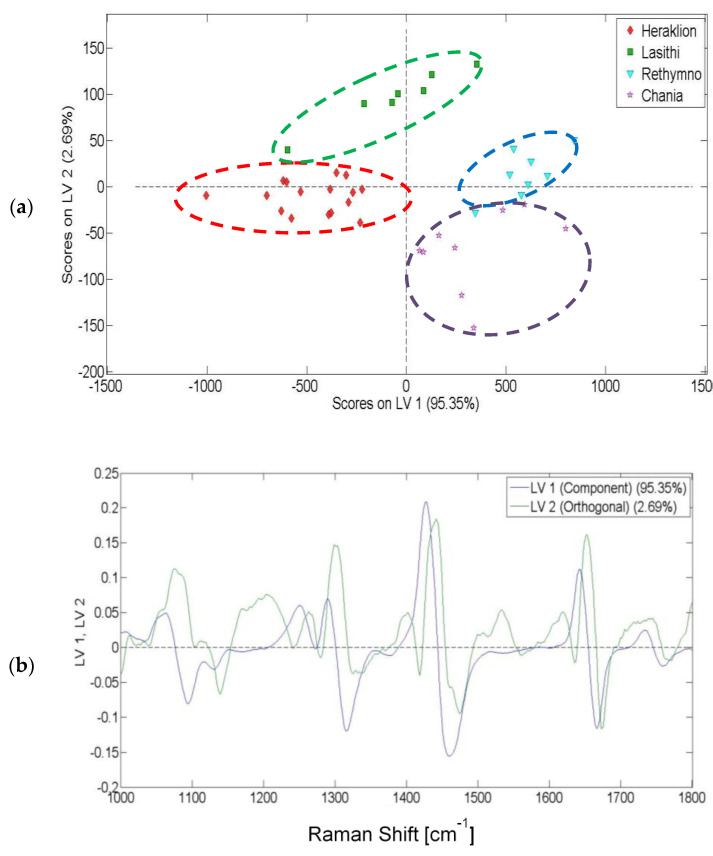
Classification of olive oils: (**a**) score plot for the discrimination of the olive oils from Crete using Raman spectroscopy; (**b**) loading plot based on Raman spectra.

For the olive oils from Crete, PLS-DA was performed on the Raman spectra from 1000 to 1800 cm^−1^. Figure 5a shows a discrimination of the olive oils produced in different prefectures of Crete (Heraklion, Lasithi, Rethymnon and Chania). It is interesting to mention that samples from Rethymno and Chania appear closer together. This may occur due to their neighboring geographical locations and as a result of similar environmental conditions. In total, 98.04% of data variance was explained by the first two latent variables (LV1 and LV2). The corresponding loading plot (Figure 5b) depicts that both the first and the second latent variable played a notable role in the discrimination of the olive oils.

**Figure 6:**
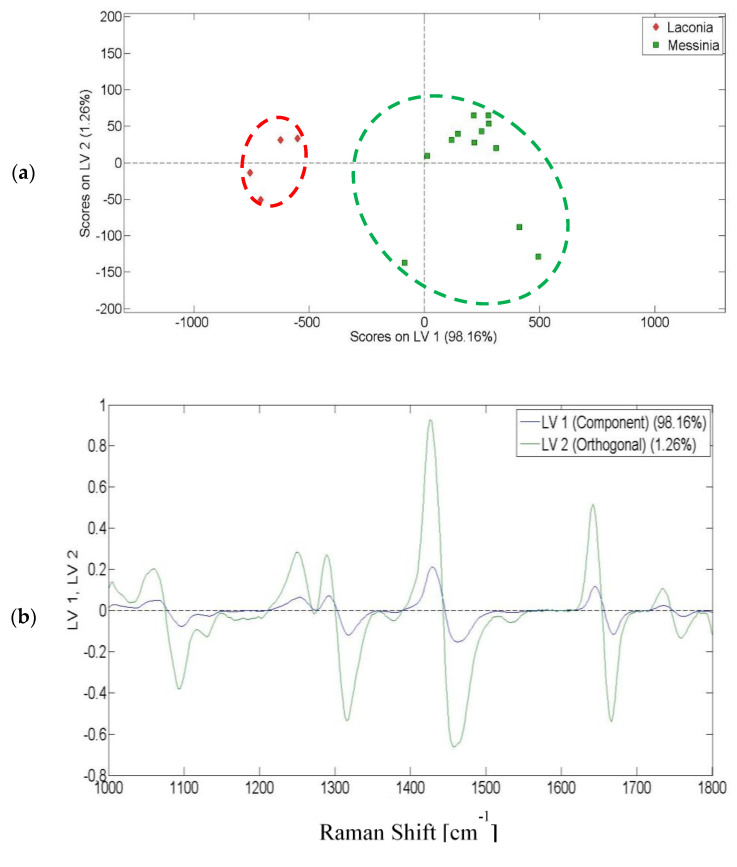
Classification of olive oils: (**a**) score plot for the discrimination of the olive oils from Peloponnese using Raman spectroscopy; (**b**) loading plot based on Raman spectra.

For the olive oils from Peloponnese, PLS-DA was performed on the Raman spectra from 1000 to 1800 cm^−1^. Figure 6a shows an unambiguous discrimination of the olive oils produced in different prefectures of Peloponnese (Laconia and Messinia). In total, 99.42% of data variance was explained by the first two latent variables (LV1 and LV2). As depicted in the relevant loading plot (Figure 6b), the second latent variable contributed to the discrimination of the olive oils.

As mentioned above, it is interesting to examine the ability of optical spectroscopy to detect the ratio of oil mixtures originating from different geographical regions. Table 4 shows the root mean squared errors of cross-validation (RMSECV) and the correlation coefficients (R^2^) for all spectral regions under examination. The application of PLS on the absorption, fluorescence and Raman spectra of the mixtures of olive oils from different geographical origins demonstrated that visible absorption spectroscopy has higher values of R^2^ in comparison to other spectroscopic techniques. Therefore, results from the visible region are presented.

**Figure 7:**
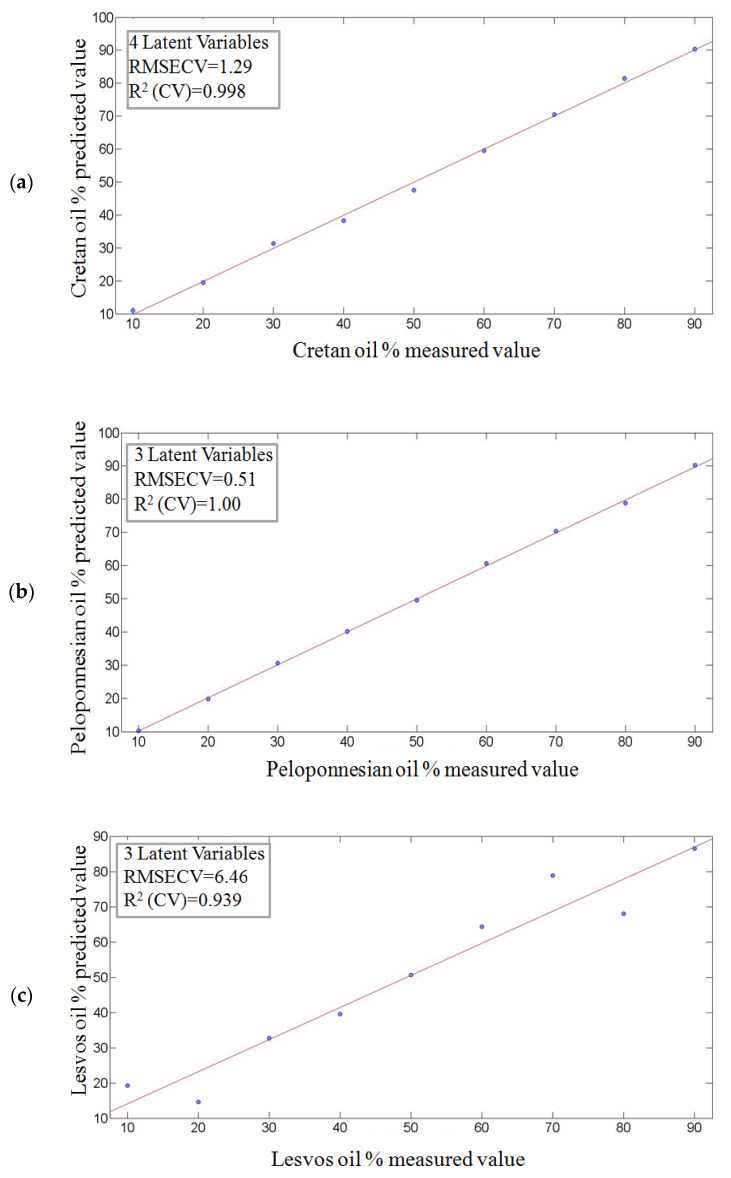
Predicted versus actual concentrations of the olive oil mixtures (% *v*/*v*) resulting from PLS model application on visible absorption spectral data (**a**) from Crete and Peloponnese, (**b**) from Peloponnese and Lesvos, and (**c**) from Crete and Lesvos.

Figure 7 presents the measured concentrations versus the predicted concentrations resulting from the application of PLS on the visible absorption spectral data. Figure 7a corresponds to the mixture of olive oils from Crete and Peloponnese and has a correlation coefficient of 0.998 and a RMSECV of 1.29. Figure 7b shows the mixture of olive oils from Peloponnese and Lesvos and has a correlation coefficient of ≈1.000 and a RMSECV of 0.51. Figure 7c depicts the mixture of olive oils from Crete and Lesvos and has a correlation coefficient of 0.939 and a root mean square error of cross-validation of 6.46.

## 3. Materials and Methods

### 3.1. Samples

Seventy-one samples of Greek olive oils were collected in the winter period of 2018–2019 from three regions of Greece, namely Crete, Peloponnese and Lesvos (Figure 1). They included 40 samples of Koroneiki variety produced in different subareas (prefectures) of Crete (16 from Heraklion, 7 from Lasithi, 8 from Rethymnon and 9 from Chania), 16 samples of Koroneiki and Kolovi varieties produced in different subareas (prefectures) of Peloponnese (12 from Messinia and 4 from Laconia) and 15 samples of Koroneiki, Kolovi and Adramitiani varieties from Lesvos. The olive oil samples were derived from both organic and conventional cultivations of similar harvesting time, altitude, irrigation scheme and fertilization. In addition, 30 binary mixtures of olive oils from different geographical origins were studied. The olive oil mixtures were prepared by blending different concentrations (10–90% *v*/*v*) of oils. The samples were stored in dark-brown glass bottles in a refrigerator at 4 °C and analyzed at room temperature.

### 3.2. Equipment and Software

Visible measurements. A Shimadzu UV-1900 spectrophotometer (Shimadzu Corporation, Kyoto, Japan) was used for collecting spectra, covering areas from 190 to 1100 nm. The spectrophotometer consisted of a dual beam optical system, two light sources (deuterium and halogen lamps), a Czerny-Turner monochromator and a silicon photodiode.

For the visible region, glass cuvettes with a 2 mm pathlength were used. Samples were measured without pretreatment and the absorbance was measured from 400 to 800 nm with a resolution of 2 nm.

Raman measurements. A mobile Raman spectrometer (HE 785, Horiba Jobin Yvon, France) was used to collect Raman spectra. The light source was a semiconductor diode laser, emitting at 785 nm. The laser was connected to the optical head of the system through a fiber optic of one-meter length and 100 μm diameter. Inside the optical head, there were necessary optics (lenses, mirrors and filters) that directed the beam and helped stimulate the sample and collected the signal. In addition, there was a led source and a multicolor camera that provided a clear image of the sample surface. A second optical fiber (2 m long and 100 µm in diameter) connected the optical head to the spectrometer (Exemplar Plus, B&W Tek, 19 Shea Way, Newark, USA), consisting of a diffraction grating monochromator and a charge-coupled device (CCD) detector. This spectrometer provided spectral coverage in the range of 98–3362 cm^−1^ (relative to the laser line) with a spectral resolution of about 8–10 cm^−1^.

In a typical Raman measurement, the olive oil sample was placed without any pretreatment in a specific sample carrier. The objective lens used provided a magnification of ×20. The samples were irradiated at a power of 68 mW, the acquisition time was 60 s and 2 scans were performed. For each sample, the measurement was repeated at different points and the average of three measurements was extracted.

NIR measurements. The Perkin-Elmer Lambda 950 spectrophotometer (Shimadzu Corporation, Kyoto, Japan) was used for collecting infrared spectra. The excitation light sources were a deuterium lamp, emitting at 190–318 nm, and a tungsten lamp, emitting at 318–2500 nm. The detector was a gridless photomultiplier tube with Peltier-controlled PbS to achieve high-performance testing across the spectral range up to 2500 nm. Measurements were performed using glass cuvettes with a 10 mm pathlength without any sample pretreatment.

Fluorescence measurements. The Horiba FluoroMax-3 (HORIBA Ltd.,Tokyo, Japan) was used for collecting fluorescence spectra. Simultaneous scanning of excitation and emission monochromators with a constant distance λ between them resulted in synchronous fluorescence spectra. The excitation wavelength ranged between 270 and 600 nm with wavelength intervals of 30, 60, 90, 120, 150, 180, 210 and 240 nm. For the fluorescence measurements, glass cuvettes with a 10 mm pathlength were used. Samples were not chemically pretreated and were placed in the front-face geometry—more specifically, at 35° to the incident beam—in order to avoid all inner filter effects.

### 3.3. Data Analysis

Data analysis was performed using MATLAB R2013b (MathWorks, MA, USA) with the PLS_Toolbox 8.1 (Eigenvector Research, Manson, WA, USA). The discrimination of the olive oil samples was achieved by partial least squares discriminant analysis (PLS-DA). PLS-DA is a supervised method. The main aim of using this method was to reduce a large number of variables to a smaller number of latent variables (LVs), enabling effective visualization and classification of data. The results are presented in the form of score plots and loading plots. A score plot is a scatter plot between two latent variables. It shows the relationship among samples by displaying how similar or different they are. A loading plot depicts the relationship among the initial variables (wavelengths or wavenumbers) and latent variables and provides the necessary information to determine which variables are responsible for the discrimination.

Partial least squares (PLS) is a technique used to model the relation between predictor matrix data and response matrix data. In this paper, PLS analysis was performed to establish a model between the actual and predicted concentrations of the olive oil mixtures from different geographical origins. The PLS models were evaluated on the basis of root mean square errors of cross-validation (RMSECV) and correlation coefficients (R2).

Both the visible and Raman spectra required pretreatment before analysis. Several pretreatments were performed, such as standard normal variate (SNV), mean center, first derivative (Savitzky–Golay) and smoothing, and it was concluded that mean center provided the best results for visible spectra. A combination of first derivative and mean center was selected to correct the baseline offset caused by sample fluorescence on the Raman spectra.

## 4. Conclusions

This study shows that visible absorption and/or Raman spectroscopy in combination with multivariate statistical analysis can distinguish olive oils produced in different geographical regions. PLS-DA models were able to classify samples from Crete, Peloponnese and Lesvos for both visible absorption and Raman spectral data. Furthermore, the application of PLS-DA to Raman spectroscopic data allowed for distinguishing samples from different sub-regions (prefectures) of Crete and Peloponnese. PLS analysis was performed to establish a model between actual and predicted concentrations of olive oil mixtures from different geographical origins. The best PLS model achieved corresponded to visible absorption spectroscopic data for the olive oil mixtures from Peloponnese and Lesvos and had a correlation coefficient of ≈1.000, with a root mean square error of cross-validation of 0.51.

Compared to other techniques used for geographical discrimination of olive oils, optical spectroscopic techniques, such as visible and Raman spectroscopies, are simpler, time saving and need no pretreatment of the sample. Moreover, a simple photometer, or even a portable Raman spectrometer, could be possibly used for on-site analysis and quality control.

## Figures and Tables

**Table 1:** Rate of successful classification (%) of the olive oil samples by partial least squares discriminant analysis (PLS-DA) for all spectral regions/methods.

Spectral Region/Method	% Successful Classification
UV 220–280 nm	84.51
UV 260–410 nm	80.28
Vis 400–800 nm	97.18
NIR 800–1600 nm	87.18
NIR 1800–2200 nm	77.46
Fluorescence	81.69
Raman	94.37

**Table 2:** PLS-DA classification of the olive oil samples using visible spectra.

	# Samples	Predicted as Crete	Predicted as Peloponnese	Predicted as Lesvos	Model Accuracy
Crete	40	40	0	0	100%
Peloponnese	16	0	15	1	93.75%
Lesvos	15	0	1	14	93.33%

**Table 3:** PLS-DA classification of the olive oil samples using Raman spectra.

	# Samples	Predicted as Crete	Predicted as Peloponnese	Predicted as Lesvos	Model Accuracy
Crete	40	39	1	0	97.50%
Peloponnese	16	2	14	0	87.50%
Lesvos	15	0	0	15	100%

**Table 4:** Root mean square errors of cross-validation (RMSECV) and correlation coefficients (R^2^) in the prediction of mixtures of olive oils from different geographical regions by PLS model.

	Crete-Peloponnese	Peloponnese-Lesvos	Crete-Lesvos
UV 220–280 nm	R^2^ = 0.777 RMSECV = 13.28	R^2^ = 0.675 RMSECV = 17.93	R^2^ = 0.965 RMSECV = 6.21
UV 260–410 nm	R^2^ = 0.931 RMSECV = 7.10	R^2^ = 0.755 RMSECV = 13.96	R^2^ = 0.968 RMSECV = 5.45
Vis 400–700 nm	R^2^ = 0.998 RMSECV = 1.29	R^2^ ≈ 1.000 RMSECV = 0.51	R^2^ = 0.939 RMSECV = 6.46
NIR 800–1600 nm	R^2^ = 0.467 RMSECV = 19.49	R^2^ = 0.687 RMSECV = 14.52	R^2^ = 0.605 RMSECV = 17.55
Fluorescence	R^2^ = 0.993 RMSECV = 2.32	R^2^ = 0.982 RMSECV = 3.52	R^2^ = 0.980 RMSECV = 3.72
Raman	R^2^ = 0.732 RMSECV = 14.26	R^2^ = 0.723 RMSECV = 14.93	R^2^ = 0.428 RMSECV = 22.21
